# Risk modeling for esophageal cancer based on adaptive Lasso and Cox regression

**DOI:** 10.3389/fonc.2025.1609540

**Published:** 2025-08-01

**Authors:** Xiaoli Li, Gaoyong Han, Yudan Yang, Enhao Liang

**Affiliations:** ^1^ Department of Oncology, the First Affiliated Hospital of Zhengzhou University, Zhengzhou, China; ^2^ School of Automation and Electrical Engineering, Zhongyuan University of Technology, Zhengzhou, China; ^3^ School of Medicine, Dalian University of Technology, Dalian, China

**Keywords:** risk modeling, esophageal cancer, post-surgical patients, adaptive Lasso, regression analysis

## Abstract

**Introduction:**

Esophageal cancer (EC) is one of the most aggressive tumor types worldwide, and malnutrition is extremely common among EC patients. By identifying EC biomarkers and conducting risk assessments on patients, more accurate diagnosis and treatment plans can be developed to prolong patients’ survival.

**Methods:**

This study developed a risk assessment model for post-surgical EC patients using clinical data from patients who underwent esophagectomy. Prognostic factors influencing survival were evaluated using Adaptive Lasso for variable selection, followed by Cox proportional hazards regression and Receiver Operating Characteristic (ROC) curve. Among multiple clinical variables, the International Normalized Ratio (INR) emerged as the most significant predictor of survival.

**Results:**

Elevated INR levels were significantly associated with improved 3-year and 5-year survival outcomes compared to the Prognostic Nutritional Index (PNI). Patients with higher INR exhibited notably better postoperative survival rates. Further analysis demonstrated that INR was significantly correlated with the final differentiation degree, final infiltration degree, and final positive/negative status of EC.

**Discussion:**

INR serves as a valuable and independent prognostic biomarker for postoperative survival assessment in EC patients. Incorporating INR into clinical risk models can enhance the accuracy of prognosis and assist clinicians in optimizing individualized therapeutic strategies for surgical EC patients.

## 1 Introduction

Esophageal cancer (EC) is a common malignant tumor of the digestive tract, ranking eighth in cancer incidence worldwide (1). In China, the incidence of EC is higher than the global average, and it is the fourth leading cause of cancer-related death (2). Esophageal cancer (EC) frequently presents with various gastrointestinal symptoms, notably dysphagia, leading to malnutrition and compromised patient conditions (3–9). The 5-year overall survival rate of EC patients remains poor, being less than 20% (10). The median survival time after diagnosis is only 11 months (11). Surgical treatment is commonly applied in the management of EC patients. However, there is risks with inflammation from surgery and required fasting further compromising the nutritional status and immunity of patients with EC. These complications not only affect the surgical outcomes of patients but also result in a poor prognosis (12–14).

The analysis of factors influencing the survival period of EC has been conducted from diverse aspects with different focuses. The main methods include factor analysis (15), linear regression analysis (16), the analytic hierarchy process (17), and the grey correlation model method (18), each having its own advantages and disadvantages. In establishing statistical models, dependent variables are not always affected by independent variables. For medical data, numerous variables are highly correlated, giving rise to statistical issues such as multiple linearity and multi-collinearity. These statistical problems introduce errors during fitting or prediction, thereby affecting the accuracy of statistical inference (19). Traditional variable selection methods are characterized by disadvantages such as slow calculation speed, poor algorithm stability, and poor model selection stability. The Lasso method was proposed to address these shortcomings (20–25). The Lasso method increases the penalty term to reduce the absolute value of the covariable coefficient to 0. It enables simultaneous variable selection and parameter estimation in the model, in contrast to traditional methods where these two steps are carried out separately. The Lasso method overcomes the drawbacks of traditional variable selection and ridge estimation and has been widely applied in various fields of modern regression analysis. To enhance the performance of the Lasso method, the adaptive Lasso algorithm and its corresponding algorithms have been proposed (24, 26–29).

In this study, based on the adaptive Lasso variable selection method, statistical techniques were utilized to analyze the factors affecting the survival time of EC patients. Cox risk regression analysis was also performed to construct the model. The established model was compared with the Prognostic Nutritional Index (PNI) in terms of 3-year and 5-year survival rates to optimize the prognostic value of PNI.

## 2 Object and analysis

### 2.1 Patient sample collection

Data of 410 patients with esophageal cancer were collected for analysis. The data were derived from clinical records of the First Affiliated Hospital of Zhengzhou University. Detailed patient information was recorded. There were 258 male patients (accounting for 62.9%), 152 female patients (accounting for 37.1%), with an age range of 45–80 years and an average age of 61.194 years. The age data were tested and shown to follow a normal distribution. Seventeen blood indicators of each patient were collected, including white blood cell (WBC) count (×10^9^/L), lymphocyte count (×10^9^/L), monocyte count (×10^9^/L), neutrophil count (×10^9^/L), eosinophil count (×10^9^/L), basophil count (×10^9^/L), red blood cell count (×10¹²/L), hemoglobin concentration (g/L), platelet count (×10^9^/L), total protein (g/L), albumin (g/L), globulin (g/L), prothrombin time (Pt, s), international normalized ratio (INR), activated partial thromboplastin time (APTT, s), thrombin time (TT, s), and fibrinogen (FIB, mg/dL).

The inclusion and exclusion criteria are listed below. Inclusion criteria: ① All patients underwent esophagectomy with lymph node dissection and had no evidence of distant metastasis at diagnosis, irrespective of neoadjuvant treatment; ②Patients were diagnosed with EC by postoperative histopathology; ③ Patients had no history of other malignancies. Exclusion criteria: ① The pathology of EC was other types, such as neuroendocrine carcinoma, lymphoma, et al.; ② Patients had incomplete follow-up records; ③ Metastatic disease was identified intraoperatively or within 1 month post-surgery.

During the data collection process, a detailed statistical analysis of missing data was carried out. It was found that there were a small number of missing values in some blood indicators, with a missing proportion of less than 5% for each. For these missing values, the Multiple Imputation by Chained Equations (MICE) method was employed. The MICE method estimates missing values multiple times based on a series of prediction models, generates multiple complete datasets, analyzes these datasets separately, and finally combines the analysis results to reduce the impact of missing values on the research findings.

### 2.2 Observed variable setting

Inflammatory response markers such as the neutrophil-lymphocyte ratio (NLR), lymphocyte-monocyte ratio (LMR), platelet-lymphocyte ratio (PLR), and prognostic nutritional index (PNI) were set as input variables. The PNI was originally established by Japanese scholars including Ono-temple and is widely used to evaluate the nutritional status of surgical patients, predict surgical risks, and assess prognosis. Its calculation formula is: PNI = serum albumin (g/L) + 5×lymphocyte count (×10^9^/L).

### 2.3 Methodology

Using patients' blood indicators as inputs and survival time as the output, the aim was to analyze the key factors influencing the survival time of EC patients. Based on the screening results of the Adaptive Lasso algorithm, SPSS 20.0 software was used for Cox proportional-hazards regression analysis, and the accuracy was evaluated according to the 3-year and 5-year survival rates of patients.

#### 2.3.1 Lasso algorithm


(1) $$\hat{\beta }=\arg\underset{\beta }{\min}||y-x\beta |{|}^{2}$$

(2) $${\sum_{t=1}^{d}\left|\beta_{i}\right|}^{2}\leq t$$

Generally, for an independent variable *y*, it may have many influencing factors called independent variable *x*. The *d*-dimensional column vector is represented by β. The symbol *t* is a compression parameter of Lasso. The important explanatory variables were expected to be obtained, which required the coefficients of individual independent variables in the model to be zero. This process was called variable selection, which was to judge the coefficient of those independent variables as zero through historical data. Thus, the variable was removed, and a sparse model was obtained. Such a sparse model was found by Lasso method.


(3) $$\hat{\beta }=\arg\underset{\beta }{\min}||y-\sum_{i=1}^{d}x_{i}\beta |{|}^{2}+\lambda \sum_{i=1}^{d}{\hat{w}}_{i}|\beta_{i}|$$

In Equation 3, λ was defined as a nonnegative regular coefficient, and $\text{λ}\sum_{i=1}^{d}{\hat{w}}_{i}\left|\beta_{i}\right|$ was called a penalty term. Equation 3 was equivalent to Equations 1 and 2. For Lasso estimation, all variables were restricted to the same parameter. In fact, λ was designed as a fixed value, which was the main reason why Lasso estimation was called biased estimation.

#### 2.3.2 Adaptive Lasso algorithm

Adaptive Lasso method was proposed in 2006, which had so-called Oracle property. The Adaptive Lasso algorithm was selected over the standard Lasso method due to its superior performance in variable selection, particularly in the presence of correlated predictors and high-dimensional covariates. By assigning adaptive weights based on initial estimates, the algorithm applies non-uniform penalization, thereby improving the accuracy of feature selection and parameter estimation. This property is especially beneficial in biomedical datasets where noise, multicollinearity, and limited sample size can impair traditional methods. For adaptive Lasso method, the penalty terms of different variables were calculated by using the least square estimation coefficient under the whole model. The large absolute value of the coefficient might be the variable in the real model, so the penalty was small. On the contrary, the small absolute value of the coefficient might not be an important independent variable, so the penalty was large. Based on this idea, the penalty function of adaptive Lasso was defined as Equations 4, 5:


(4) $$\hat{\beta }=\arg\underset{\beta }{\min}||y-\sum_{i=1}^{d}x_{i}\beta |{|}^{2}+\lambda_{n}\sum_{i=1}^{d}{\hat{w}}_{i}|\beta_{i}|$$

(5) $$\hat{w}=\frac{1}{|\hat{\beta }(ols){|}^{\gamma }},\;\gamma >0$$

The formula can be defined as an adaptive Lasso, where each penalty item was given a different weight according to the size of the estimated parameters. The ordinary least squares of is represented by $\hat{\beta }\left(ols\right)$ , and $\hat{\beta }$ is the root uniform estimate of *β*. γ is a random number with greater than 0. The parameter λ of the penalty function was not defined as a fixed value, but it was varied according to the value of each variable. The model will select more variables than Lasso regression without affecting the fitting effect, and the over compression of parameters can be prevented. In the implementation of the Adaptive Lasso algorithm, the regularization parameter λ was selected using 10-fold cross-validation to minimize prediction error. The weight exponent γ was set to 1, which provides a good balance between model sparsity and stability.

#### 2.3.3 Model stability test

To ensure the stability and reliability of the constructed model, the 10-fold cross-validation method was used to evaluate the model. The specific operation is as follows: The original dataset was randomly divided into 10 subsets of similar sizes. For each round of analysis, 9 of these subsets were selected as the training set for model construction and training, while the remaining 1 subset was used as the test set to evaluate the model's performance. This process was repeated 10 times, ensuring each subset was used as a test set exactly once, thereby reducing bias and improving the reliability of performance estimates. Finally, 10 sets of model evaluation results were obtained. By calculating the mean and standard deviation of various indicators (such as the prediction accuracy of 3-year and 5-year survival rates, the area under the ROC curve, etc.) of these 10 evaluation results, the stability of the model was measured. If the standard deviation of the performance indicators is small, it indicates that the model performs stably on different datasets and has high reliability.

### 2.4 Follow-up

The First Affiliated Hospital of Zhengzhou University developed a systematic follow-up plan. Professional staff were randomly assigned to conduct telephone follow-up and regular outpatient follow-up. The follow-up period spanned 6–140 months to comprehensively track the survival status of patients. Patients lost to follow-up within 6 months post-surgery were excluded from the final analysis to minimize bias and maintain the robustness of our results. Follow-up was considered complete if patients had at least 6 months of postoperative survival data. Within 1 month after treatment, patients underwent esophagography and chest CT to evaluate short-term clinical effects. In the first 2 years after surgery, patients were followed up every 3 months; after 2 years, they were followed up every 6 months. During each follow-up, detailed information about the patient's survival status, disease recurrence, and other relevant clinical information was recorded.

## 3 Results

Experiment 1: Using 17 blood indicators as input variables and survival time as the output variable, the Adaptive Lasso algorithm was applied, and the results are shown in **Table 1**.

**Table 1:** Analysis of modeling results of adaptive Lasso and Cox regression.

Experiment	Screening of variables	Cox regression (State)	Sig
Experiment 1	INR、APTT	-0.969*INR+0.02*APTT(<5 years)	0.014
Experiment 2	INR	-0.861*INR (death)	0.038
Experiment 3	INR	-0.861*INR (death)	0.038
Experiment 4	INR、APTT、PLR	0.002*PLR-1.094*INR+0.02*APTT (death)	0.001
Experiment 5	PLR	0.02*PLR (death)	0.035

Experiments 2-4: Considering the possible complex relationships among variables, different combinations of blood indicators (removing lymphocyte-related indicators and adding other variables) and PNI were used as input variables, and survival time was used as the output variable. The Adaptive Lasso algorithm was applied, and the results are also shown in **Table 1**. In the experimental design process, the removal of lymphocyte-related indicators was based on preliminary exploratory analysis. It was found that the newly added variables had a strong linear relationship with the lymphocyte indicators in the blood. This collinearity might interfere with the variable selection and parameter estimation results of the model, affecting the accuracy and stability of the model. Therefore, in subsequent experiments, lymphocyte-related indicators were removed to optimize the model construction.

Experiment 5: Using 17 blood indicators, and PLR as input variable and survival time as the output variable, the Adaptive Lasso algorithm was applied, and the results are shown in **Table 1**.

The variables screened from the above 5 experiments were used as the input variables for Cox risk regression, and survival time was used as the output variable. The corresponding analysis results are shown in **Table 1**. Given the large number of experimental combinations tested, **Table 1** presents only the regression results that showed statistically significant associations between the selected variables and survival outcomes. The INR variable was not selected only in Experiment 5, and the significance correlation test value of risk regression was set at 0.05.

**Figure 1:**
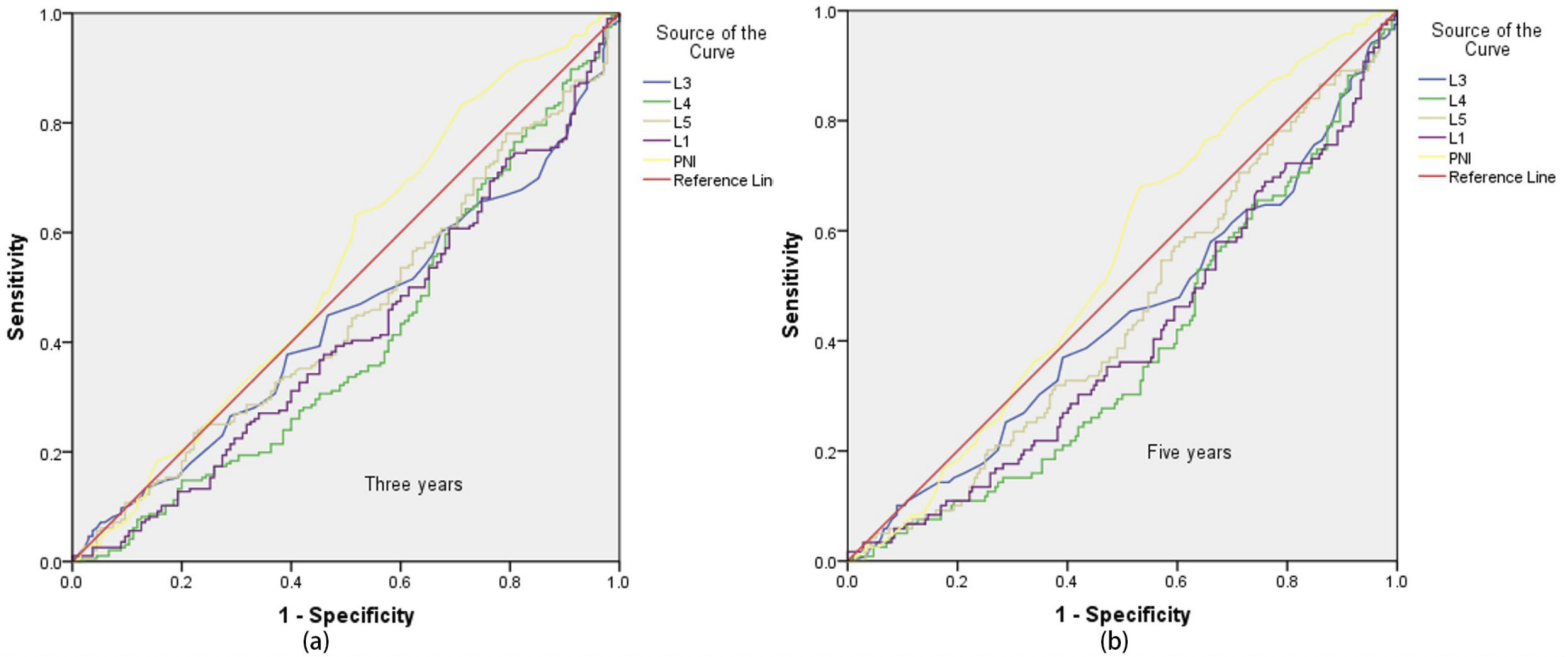
ROC curve with 3-year and 5-year survival as the critical value. Sub-figures (**a**) and (**b**) show the ROC curves for 3-year and 5-year survival, respectively.

Receiver Operating Characteristic (ROC) curves were plotted with a 3-year survival period as the critical value (**Figure 1a**). It was found that only the area under the PNI curve was greater than 0.5. When the ROC curve was plotted with a 5-year survival period as the critical value (**Figure 1b**), the area under the PNI curve was also greater than 0.5. However, the statistical analysis of the Cox regression model showed that none of these 5 models were statistically significant under the current settings (detailed data are shown in **Tables 2** and **3**). It is important to note that statistical significance merely indicates that the model performs better than random guessing. Therefore, statistical significance should not be equated with clinical utility, and the predictive value of INR as a standalone factor should be interpreted with caution.

**Table 2:** Area under ROC curve with 3-year survival as critical value.

Test result variable(s)	Area	Std. error	Sig	Asymptotic 95% confidence interval	
				Lower bound	Upper bound
L3	0.441	0.032	0.068	0.379	0.503
L4	0.404	0.032	0.003	0.341	0.467
L5	0.452	0.032	0.141	0.390	0.515
L1	0.414	0.032	0.008	0.352	0.476
PNI	0.541	0.033	0.203	0.477	0.606

**Table 3:** Area under the curve with 5-year survival as the critical value.

Test result variable(s)	Area	Std. error	Sig	Asymptotic 95% confidence interval	
				Lower bound	Upper bound
L3	0.439	0.033	0.065	0.373	0.504
L4	0.382	0.032	0.000	0.320	0.444
L5	0.448	0.032	0.120	0.385	0.512
L1	0.400	0.032	0.003	0.337	0.463
PNI	0.543	0.032	0.198	0.480	0.605

Given that the above 5 models did not show statistical significance in the analysis of the 3-year and 5-year survival rates of EC patients, based on the preliminary analysis results of the Adaptive Lasso experiments, experiment was further carried out. ROC curve analyses of INR were performed using 150, 200, 250, and 330 cases of data respectively. The results showed that when 3-year and 5-year survival were used as the critical values, the areas under the ROC curves of all data were greater than 0.5 (specific data are shown in **Table 4**), indicating a close association between INR and the survival time of patients.

**Table 4:** Comparison between INR and PNI of different grouping data.

Status	INR area under the curve (AUC)				PNI area under the curve (AUC)			
Status	150	200	250	330	150	200	250	330
3-year	0.564	0.550	0.571	0.559	0.507	0.573	0.551	0.541
5-year	0.575	0.577	0.591	0.561	0.485	0.543	0.543	0.543

A Cox regression model was established with INR as the input variable and survival time as the output variable. In this study, “lifetime” refers to the time from surgical treatment to death from any cause. “Survival rate” refers to the percentage of patients who remained alive at predefined time points (e.g., 3-year and 5-year survival). The significant correlation between the model and the survival rate is shown in **Table 5** (P = 0.015), indicating that the model has certain predictive values. The total data were divided into an experimental group (250 cases) and a test group (160 cases). 3-year and 5-year survival analyses of PNI and INR were performed (detailed results are shown in **Tables 6** and **7**). The threshold of PNI was determined to be 46.75 and that of INR was 0.905 based on the ROC curve. This approach was chosen to ensure optimal sensitivity and specificity for predicting 3-year and 5-year survival outcomes. Comparative analysis showed that in the high-concentration case, the 3-year and 5-year survival rates of the INR group were higher than those of the PNI group.

**Table 5:** Omnibus tests of model coefficients.

-2 Log likelihood	Overall (Score)		
	Chi-square	df	Sig.
2616.533	5.913	1	0.015

**Table 6:** Comparison of PNI and INR survival rates in the experimental group.

Lifetime	INR (0.905)		PNI (46.75)	
	High-INR	Low-INR	High-PNI	Low-PNI
Alive	21 (32.81%)	44 (23.66%)	53 (27.75%)	12 (20.34%)
Death	43 (67.19%)	142 (76.34%)	138 (72.25%)	47 (79.66%)
>3 years	48 (75.00%)	106 (56.99%)	129 (67.54%)	25 (42.37%)
<3 years	16 (25.00%)	80 (43.01%)	62 (32.46%)	34 (57.63%)
>5 years	31 (48.44%)	68 (36.56%)	85 (44.50%)	14 (23.73%)
<5 years	33 (51.56%)	118 (63.44%)	106 (55.50%)	45 (76.27%)

**Table 7:** Comparison of survival rate between PNI and INR in test group.

Lifetime	INR (0.905)		PNI (46.75)	
	High-INR	Low-INR	High-PNI	Low-PNI
Alive	14 (42.42%)	26 (20.47%)	34 (29.31%)	6 (13.64%)
Death	19 (57.58%)	101 (79.53%)	82 (70.69%)	38 (86.36%)
>3 years	23 (69.70%)	67 (52.76%)	68 (58.62%)	22 (50.00%)
<3 years	10 (30.30%)	60 (47.24%)	48 (41.38%)	22 (50.00%)
>5 years	16 (48.48%)	38 (29.92%)	42 (36.21%)	12 (27.27%)
<5 years	17 (51.52%)	89 (70.08%)	74 (63.79%)	32 (72.73%)

Further analysis by chi-square test found that both the INR concentration classification and the PNI concentration classification were significantly correlated with the survival classification P<0.05, detailed data are shown in **Table 8**), further verifying the importance of INR and PNI in evaluating the survival prognosis of esophageal cancer patients.

**Table 8:** Comparison of chi-square test between PNI and INR.

Lifetime	INR (0.905)				PNI (46.75)			
	High-INR	Low-INR	Chi-square value	Sig	High-INR	Low-INR	Chi-square value	Sig
Alive	35 (36.08%)	70 (22.36%)			87 (28.34%)	18 (17.48%)		
Death	62	243	7.315	0.006	220 (71.66%)	85 (82.52%)	4.777	0.018
>3 years	71 (73.20%)	173 (55.27%)			197 (64.17%)	47 (45.63%)		
<3 years	26	140	9.874	0.001	110 (35.83%)	56 (54.37%)	11.000	0.001
>5 years	47 (48.45%)	106 (33.87%)			127 (41.37%)	26 (25.24%)		
<5 years	50	207	6.737	0.007	180 (58.63%)	77 (74.76%)	8.573	0.002

**Figure 2:**
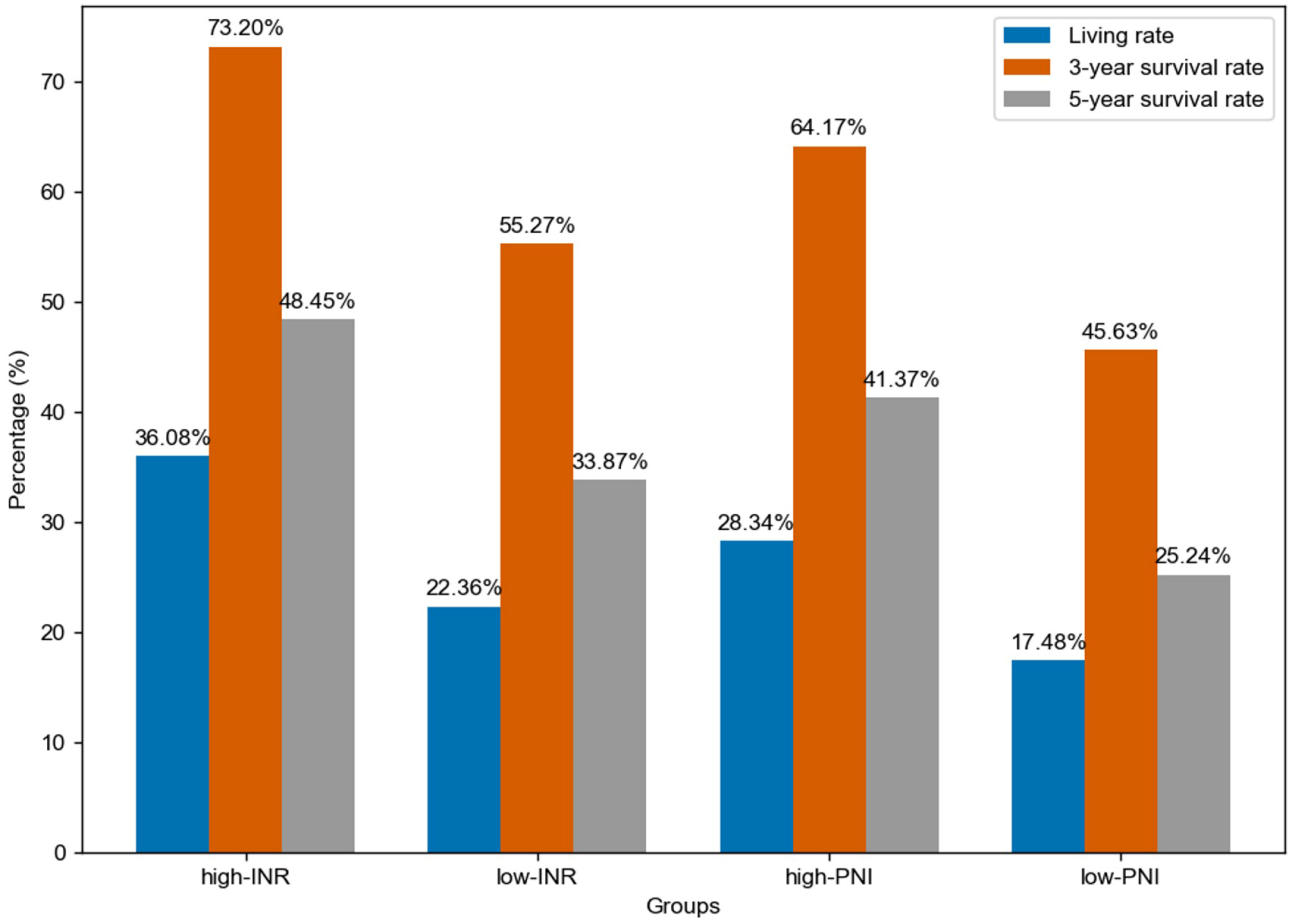
Comparison of survival rates between INR and PNI.

From a biological perspective, INR, as a crucial indicator reflecting blood coagulation function, plays a vital role in the tumor microenvironment. As shown in **Figure 2**, for different groups (high-INR, low-INR, high-PNI, low-PNI), the 3-year survival rate of the high-INR group was 73.20%, the 5-year survival rate was 48.45%, and the living rate was 36.08%; the 3-year survival rate of the low-INR group was 55.27%, the 5-year survival rate was 33.87%, and the living rate was 22.36%; the 3-year survival rate of the high-PNI group was 64.17%, the 5-year survival rate was 41.37%, and the living rate was 28.34%; the 3-year survival rate of the low-PNI group was 45.63%, the 5-year survival rate was 25.24%, and the living rate was 17.48%.

## 4 Discussion

**Figure 3:**
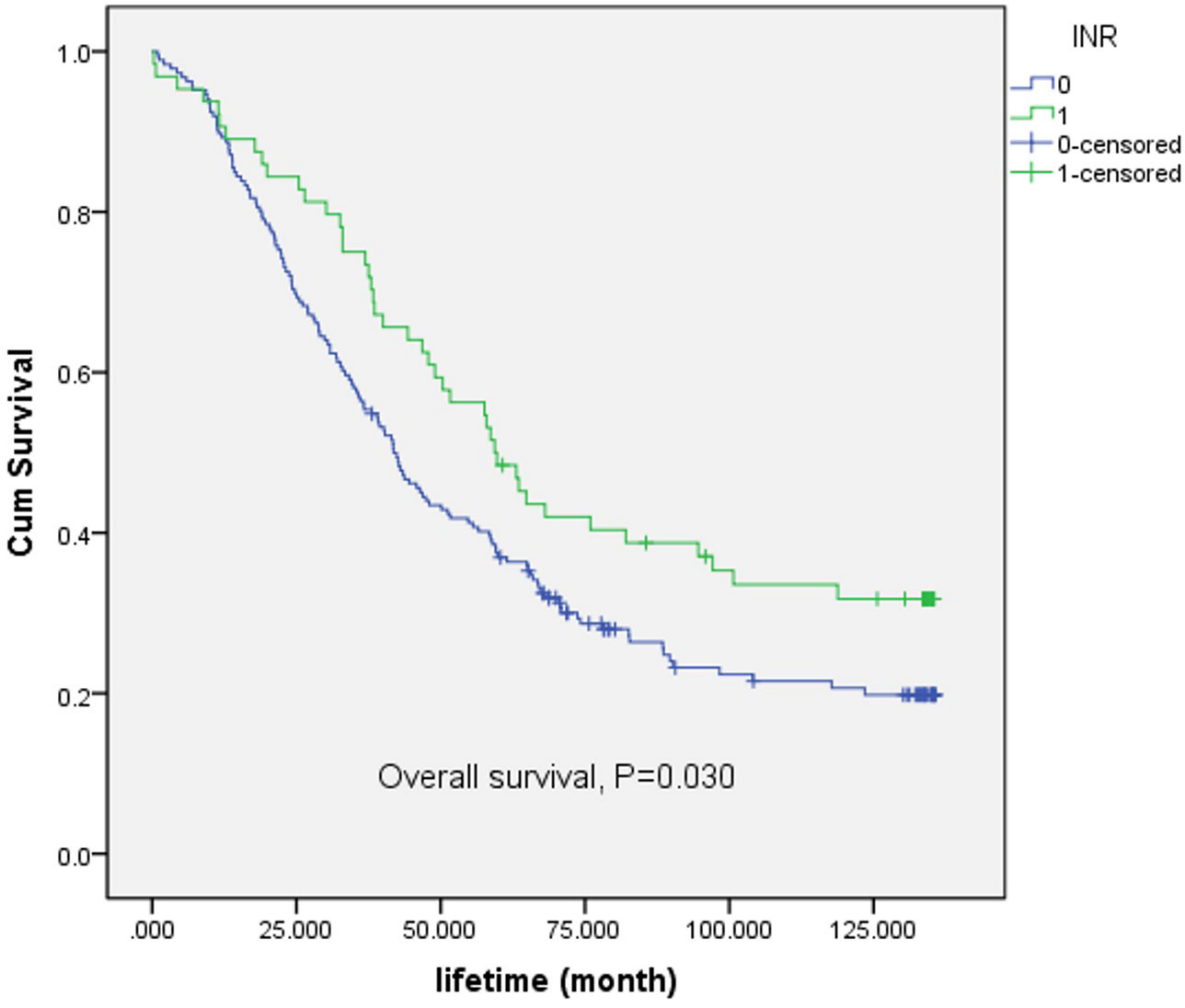
Survival analysis of INR. Low-INR is expressed by 0, high-INR is expressed by 1.

Following the comparison of the survival rates associated with INR (International Normalized Ratio) and PNI (Prognostic Nutritional Index), further exploration of INR is warranted. A Kaplan-Meier (K-M) survival curve was plotted, as depicted in **Figure 3**. For the overall survival rate, there was significant difference between low-INR and high-INR (p = 0.030). The results indicated a significant correlation between INR classification and survival time.

Based on the clinicopathological analysis of EC patients, clinicopathological characteristics were classified and cross-tested according to the high and low concentrations of INR, as presented in **Table 9**. Regarding staging of patients, TNM Staging was evaluated by postoperative histopathology. The results in **Table 9** indicated that INR was significantly correlated with final differentiation, final infiltration, and final positive/negative status. When the test level was set at 90%, a significant correlation was also observed with the latest TNM stage of the eighth edition.

**Table 9:** Clinicopathological features of patients (INR).

Clinicopathological features	INR >0.9050		INR<0.9050		T		Sig
	N	%	N	%	N	%	
Gender							0.246
Male	44	68.75%	117	62.90%	161	64.40%	
Female	20	31.25%	69	37.10%	89	35.60%	
Diagnostic age							0.237
> 60	28	43.75%	93	50.00%	121	48.40%	
< 60	36	56.25%	93	50.00%	129	51.60%	
Final tumor site							0.992
Upper Thoracic Segment	9	14.06%	38	20.43%	47	18.80%	
Inferior Thoracic Segment	13	20.31%	25	13.44%	38	15.20%	
Middle Thoracic Segment	42	65.63%	123	66.13%	165	66.00%	
Final degree of differentiation							<0.001
Highly Differentiated	7	10.94%	10	5.38%	17	6.80%	
Low Differentiation	22	34.38%	78	41.94%	100	40.00%	
Middle Differentiation	35	54.69%	98	52.69%	133	53.20%	
Final infiltration degree							0.032
Muscular Layer (Superficial Muscular Layer)	11	17.19%	12	6.45%	23	9.20%	
Muscular Layer (Deep Muscular Layer)	8	12.50%	38	20.43%	46	18.40%	
Fibrous Membrane	34	53.13%	119	63.98%	153	61.20%	
Mucous Layer	2	3.13%	2	1.08%	4	1.60%	
Mucous Layer (Muscularis Mucosae)	3	4.69%	3	1.61%	6	2.40%	
Submucosa	6	9.38%	12	6.45%	18	7.20%	
Clean / unclean cutting edge							0.518
Net	63	98.44%	181	97.31%	244	97.60%	
Not Clean	1	1.56%	5	2.69%	6	2.40%	
Final positive / Negative							0.040
Positive	27	42.19%	104	55.91%	131	52.40%	
Negative	37	57.81%	82	44.09%	119	47.60%	
The latest TNM staging of the eighth edition							0.062
Stage I	10	15.63%	11	5.91%	21	8.40%	
Stage II	29	45.31%	77	41.40%	106	42.40%	
Stage III	21	32.81%	83	44.62%	104	41.60%	
Stage IV A	4	6.25%	15	8.06%	19	7.60%	

**Figure 4:**
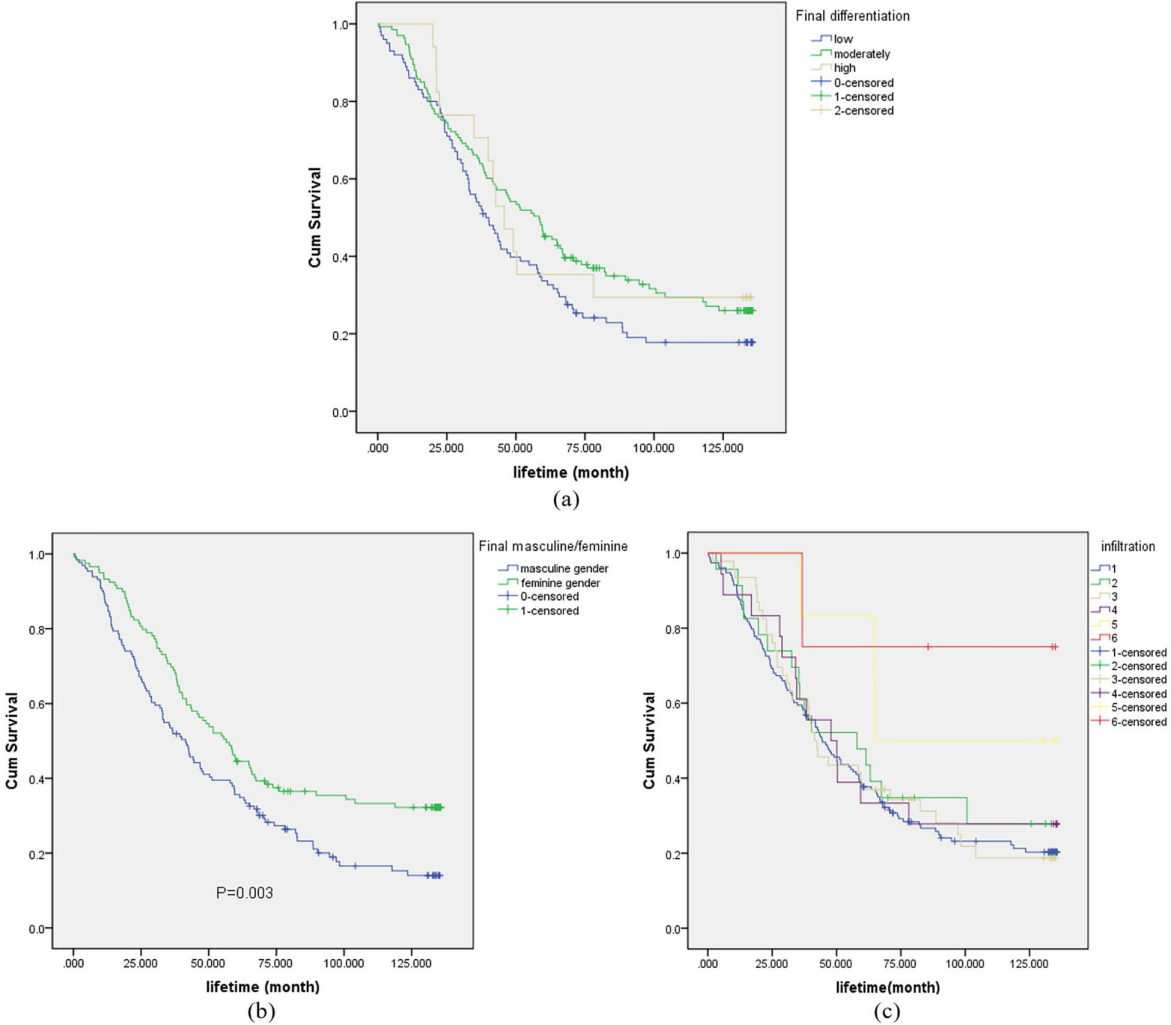
Survival curve for final differentiation degree (sub-figure **a**), final positive/negative (sub-figure **b**) and final infiltration degree (sub-figure **c**).

Survival curves for final differentiation degree, final infiltration degree, and final positive/negative status were plotted, as shown in **Figure 4**. In **Figure 4a**, the survival rates of patients with high-grade differentiation exhibited greater fluctuations, while those with medium-grade and low-grade differentiation were relatively stable. Notably, the survival rate of patients with medium-grade differentiation was higher than that of those with low-grade differentiation. As depicted in **Figure 4b**, the survival rate of positive patients was lower than that of negative patients. In **Figure 4c**, 1–6 represent the fibrous membrane, muscular layer (superficial muscle layer), muscular layer (deep muscle layer), submucosa, mucosal layer (mucosal muscle layer), and mucosal layer, respectively.

**Figure 5:**
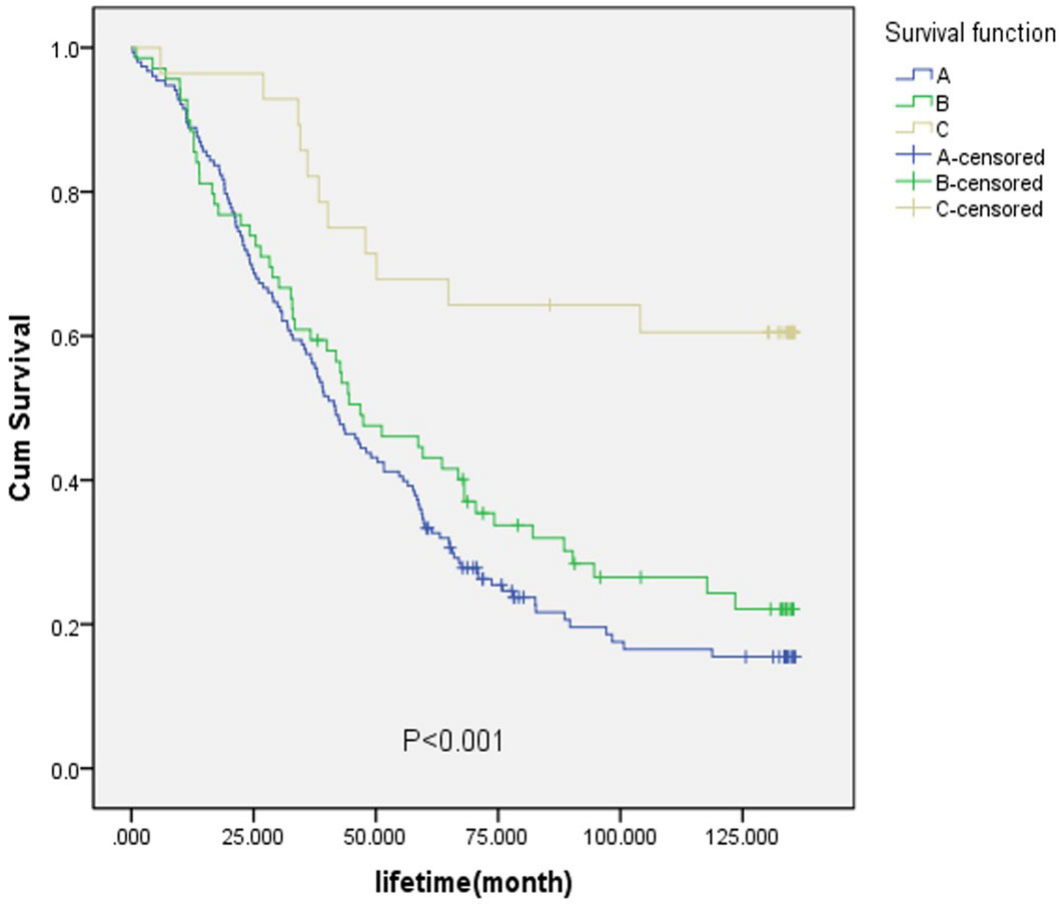
Ultimate infiltration degree graded survival curve.

For the purpose of analysis, the fibrous membrane was designated as A, the muscular layer (superficial muscle layer) and the muscular layer (deep muscle layer) as B, and the submucosa, mucosal layer (mucosal muscle layer), and mucosal layer as C. As shown in **Figure 5**, the survival rate of layer C was the highest, with a significant value of p<0.001. When considering the TNM staging of EC and the degree of tumor invasion, it was evident that the survival rate of patients with early-stage EC was higher than that of patients with late-stage EC.

**Figure 6:**
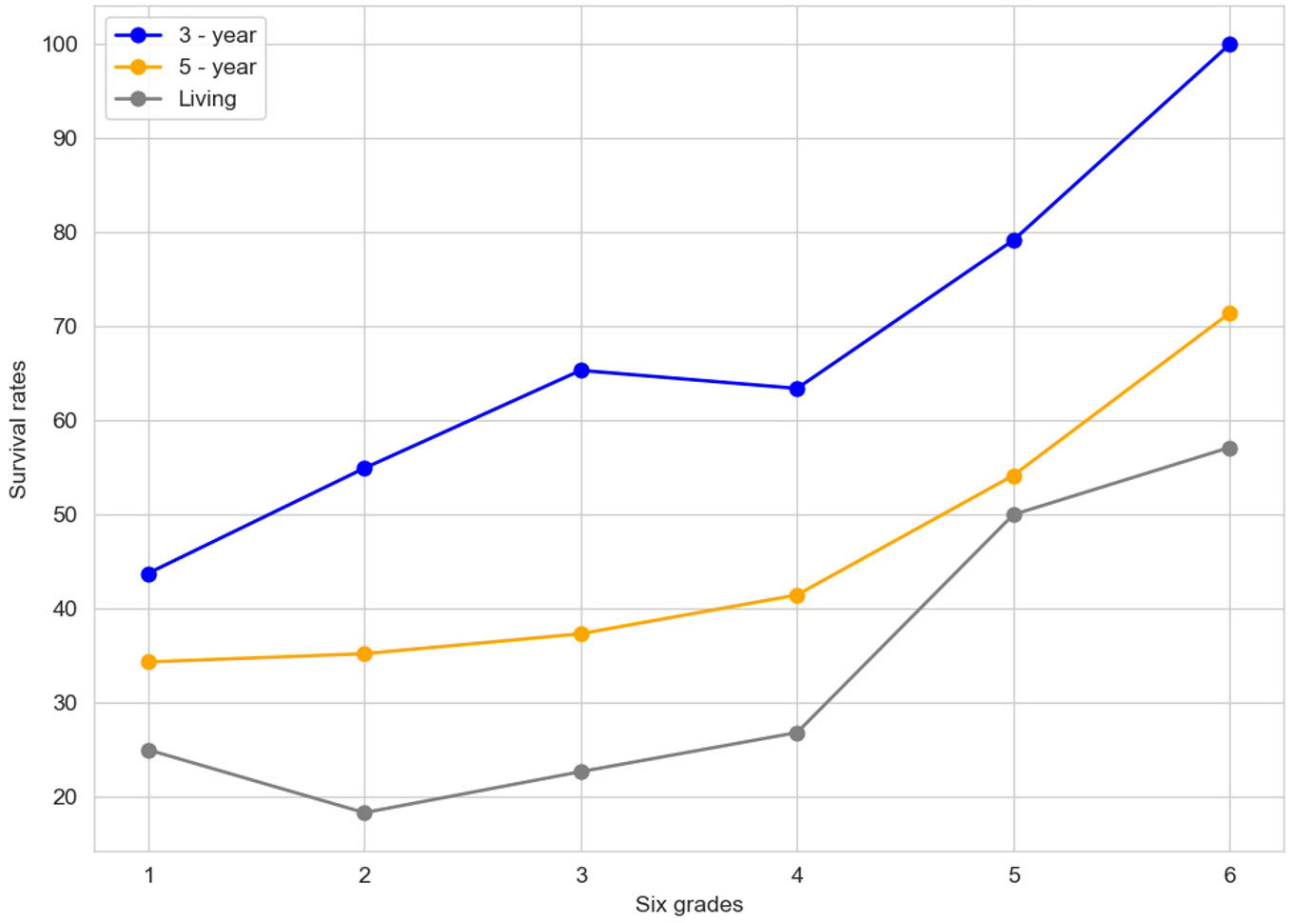
Lifetime grade line chart.

The "Contrast figure" (**Figure 6**) illustrates the survival rates across six distinct grades, with lines representing the 3-year survival rate (in blue), the 5-year survival rate (in orange), and the living rate (in gray). The six-grade risk model presented in this study integrates key prognostic variables identified through adaptive Lasso and Cox regression. This semi-quantitative grading system was designed based on a combination of data-driven selection and clinical interpretability.

In the realm of constructing a risk model for esophageal cancer, as previously elaborated, several critical factors come into play. Variables such as the INR and the PNI have been central to our analysis. Through methodologies like the Adaptive Lasso and Cox regression analyses, INR has emerged as a key determinant significantly associated with patient survival time.

The six grades depicted in this figure likely encapsulate different strata of risk factors or clinicopathological features. For example, in the clinicopathological analysis of EC patients, elements such as final differentiation, final infiltration, and final positive/negative status, which exhibit a significant correlation with INR, could potentially underpin the definition of these grades.

The construction of these risk grades is likely a multifaceted process. INR, given its strong association with pivotal clinicopathological aspects like differentiation, infiltration, and positive/negative status, could play an important role. Generally, a higher INR is often associated with a more favorable risk grade. This is because a higher INR may align with better differentiation, reduced infiltration, and a negative status, all of which are typically linked to improved survival prospects. Additionally, other variables, such as PNI and various blood indicators initially considered in the model - building process, could contribute to the nuanced determination of these risk grades.

Visually, as we move from grade 1 to grade 6, the upward trend in the 3-year and 5-year survival rates, as well as the living rate, suggests that higher grades may signify a more favorable prognosis. This graphical representation provides a crucial visual cue for understanding how survival outcomes vary across different risk-grade categories, thereby facilitating the refinement and validation of the esophageal cancer risk model. It serves as a valuable tool for further research and clinical decision-making, enabling a more precise assessment of patient prognosis based on these risk-grade classifications.

It should be pointed out that although INR demonstrated statistical significance in survival prediction, the corresponding AUC values remained modest (mostly below 0.6), indicating limited discriminatory power. The INR-based model is intended as a complementary tool for risk stratification rather than a stand-alone decision-making metric. Due to the complexity and heterogeneity of EC prognosis, especially in surgical patients, even modest gains in discrimination may provide meaningful insights when combined with clinical judgment. This finding suggests that while INR holds prognostic value, it may be insufficient as a standalone marker. Future work should explore composite models that integrate INR with additional clinical, pathological, or molecular features to improve risk stratification performance.

## 5 Conclusion

In conclusion, this study aimed to construct a risk model for esophageal cancer by integrating the Adaptive Lasso and Cox regression models. Through a comprehensive analysis of 410 esophageal cancer patients' data, several significant findings were obtained.

The INR emerged as a key variable closely associated with the survival time of esophageal cancer patients. Multiple experimental analyses, including those using the Adaptive Lasso algorithm, consistently identified INR as a crucial factor. Clinicopathological analyses further revealed that INR was significantly correlated with important aspects such as final differentiation, final infiltration, and final positive/negative status of the tumors. This indicates that INR not only reflects the patient's coagulation function but also has profound implications for understanding the biological behavior of esophageal cancer.

Compared with the PNI, INR demonstrated unique advantages in predicting the survival prognosis of esophageal cancer patients, especially in high-concentration scenarios where the 3-year and 5-year survival rates of the INR group were higher. The Kaplan-Meier survival curve analysis also confirmed the significant correlation between INR classification and survival time, providing strong evidence for its role in prognosis evaluation.

However, it is important to acknowledge the limitations of this study. The sample size, although relatively large, may still have limitations in fully representing the diverse characteristics of the entire esophageal cancer patient population. The findings may not generalize to populations with different demographic or pathological characteristics, such as those in Western countries where adenocarcinoma predominates. Future research should strive to include a more extensive and diverse sample from different geographical regions and ethnic groups to enhance the generalizability of the findings. Moreover, due to the retrospective nature of the study and constraints in follow-up data, we used all-cause mortality rather than cancer-specific death as the endpoint. As such, the model predicts overall survival, which may be influenced by non-cancer-related comorbidities. Additionally, the current study focused mainly on blood-related indicators. Incorporating multi-omics data, such as gene expression and proteomics information, in future investigations could offer a more in-depth understanding of the molecular mechanisms underlying esophageal cancer development and further optimize the risk model.

Overall, this study has made a significant contribution by identifying INR as a potential biomarker for esophageal cancer risk assessment. The findings provide valuable insights for clinical practice, potentially enabling more accurate prognosis prediction and personalized treatment strategies for esophageal cancer patients. Continued research in this area is expected to further refine the risk model and improve the overall management of esophageal cancer.

## Data Availability

The raw data supporting the conclusions of this article will be made available by the authors, without undue reservation.
